# The ‘empty choice’: A sociological examination of choosing medical research participation in resource-limited Sub-Saharan Africa

**DOI:** 10.1177/0011392115590093

**Published:** 2015-07-13

**Authors:** Patricia Kingori

**Affiliations:** University of Oxford, UK

**Keywords:** Choice, frontline workers, informed consent, research participation, Sub-Saharan Africa, Afrique sub-saharienne, choix, consentement éclairé, participation à une recherche, travailleurs de première ligne

## Abstract

This article explores the views of frontline research staff in different Sub-Saharan African contexts on the notion of choice in biomedical research. It argues that the current emphasis on individual choice, in the conduct of biomedical research, ignores significant structural and contextual factors in resource-limited settings. These factors severely constrain individual options and often make biomedical research enrolment the most amenable route to healthcare for the world’s poorest. From the position of frontline research staff, local contextual factors and structural issues narrowly frame the parameters within which many prospective participants are asked to choose, to such an extent that individuals are effectively presented with an ‘empty choice’. The article draws on ethnographic and interview data and insights gained through graphic elucidation techniques. It demonstrates that for frontline research staff, macro-level structural factors and their bearing on everyday realities shape what choice in biomedical research participation means in practice.

## Résumé

Cet article explore les opinions de chercheurs de première ligne travaillant dans différents contextes sub-sahariens sur la notion de choix en recherche biomédicale. L’argument est que l’accent placé actuellement sur le choix individuel ne tient pas compte des facteurs structuraux et contextuels significatifs dans un cadre où les ressources sont limitées. Ces facteurs limitent sévèrement les options individuelles et font que la participation à une recherche biomédicale est souvent la voie la plus accommodante pour les pauvres qui souhaitent avoir accès aux soins de santé. Du point de vue des chercheurs de première ligne, des facteurs contextuels et des problèmes structuraux encadrent étroitement les paramètres au sein desquels de nombreux participants prospectifs sont invités à choisir, si bien qu’ils sont en fait exposés à un « choix illusoire ». L’article se fonde sur des données ethnographiques, des données recueillies en entrevue et des indications obtenues par le biais de techniques d’élucidation graphique. Il démontre que pour les chercheurs de première ligne, des facteurs macrostructuraux et leur impact sur les réalités quotidiennes influencent ce que le choix de participer à une recherche biomédicale signifie en pratique.

## Resumen

Este artículo explora las opiniones del personal de investigación de primera línea en los diferentes contextos del África subsahariana respecto de la noción de elección en la investigación biomédica. Se argumenta que el énfasis actual en la elección individual ignora los factores estructurales y contextuales significativos en entornos con recursos limitados. Estos factores limitan severamente las opciones individuales y hacen de la inscripción en la investigación biomédica la ruta más viable para que los pobres tengan acceso a la asistencia sanitaria. Desde la posición del personal de investigación de primera línea, los factores contextuales y los problemas estructurales enmarcan estrictamente los parámetros dentro de los cuales se les pide elegir a muchos posibles participantes, hasta el punto en que de hecho se les presenta una ‘elección vacía’. El documento se basa en datos y conocimientos etnográficos y en entrevistas obtenidas a través de técnicas de elucidación gráficas. Esto demuestra que para el personal de investigación de primera línea, los factores estructurales a nivel macro y su relación con la realidad cotidiana determinan lo que significa en la práctica la elección en la participación de la investigación biomédica.

## Introduction

Biomedical research is increasingly conducted in locations with under-resourced health systems. In these contexts, research offers its participants treatment and access to healthcare, which is otherwise scarce, either as ad hoc ancillary care, or as a way of ‘sharing benefits’ (Hayden, 2007). Biomedical research differs from healthcare in its intention to benefit future populations rather than individual research participants. While it aims not to harm participants, biomedical research constructs specific obligations and rights to health, providing access to drugs and support exclusively to individual research participants. At the same time, the quality, range and ease of healthcare access provided by biomedical research often stand in stark contrast to the resources available in over-stretched public health systems.

Despite the glaring disparity in healthcare services provided by public health and biomedical research institutions, prospective participants in resource-poor localities are expected to overlook these differences in their decision to be involved in biomedical research. The 24-hour access to highly trained clinicians, administering regulated medicines, in calm and comfortable air-conditioned clinical spaces should be irrelevant to considerations made about research participation. Potential research participants are encouraged to make an objective choice, ‘unbiased’ by the differences in healthcare that they observe, experience and which can make the difference between life and death.

It is argued in this article that while biomedical research often involves the rhetoric of choice, or the freedom for individuals to choose to participate in research, macro-economic and structural factors frame the options and context in which many prospective participants in Sub-Saharan locations are asked to make such choices. Hence, for those who prioritise their health, the lack of viable options effectively represents an ‘empty choice’. The discourse of choice creates an illusion of individual freedom and power, without consideration of structural factors which constrain those choices. Drawing on interviews and observations conducted in a variety of resource-limited settings in Sub-Saharan Africa, the views and position of frontline research staff are explored in this article. The data presented complement examinations of choice in patients and research participants in a variety of European and North American contexts. In examining their views, this article explores how choice has been interpreted and the challenges of promoting choice when structural factors constrain individual options.

### The logic of choice

In the last 30 years there has been considerable sociological attention paid to the relationship between neoliberal policies and healthcare in numerous countries and contexts. This literature has elucidated the negative consequences of macro-economic policies and priorities which have diverted resources from public to private institutions. This process has allowed for healthcare provision to be dictated by market forces, and the individualisation of health for patients and healthcare staff. In these accounts, the patient as consumer of health services has had their well-being compromised while assuming increasing responsibility (and burdens) for their ‘chosen’ course of action. Simultaneously, health professionals have witnessed employment insecurity, the devaluation of their caring capacity and low morale if they ‘choose’ to work within the public sector, in which the ‘logic of choice’ has prevailed (Mol, 2008).

The ‘logic of choice’, Mol argues, is based on the liberal principle that people should be permitted to make their own choices, if no harm is caused to others. This logic is argued to have reconfigured healthcare and ways of understanding patients and patient interactions (Clarke, 2010). For Mol (2008) the logic of choice has two main manifestations: consumer or market choice and citizen choice. Market choice transforms patients into customers and citizenship choice is critiqued for regarding patients solely as citizens, and healthcare as akin to rights and duties governable by contracts. For Mol, choice operates in opposition to ideas of paternalism and care in health service provision.

Writing in 2010, Nordgren draws on Michel Foucault’s concepts of discursive formation and subjectification (1972, 1988) and Judith Butler’s (1993) work on performativity in his description of the discourse of choice as ‘empty words’. Nordgren summarises the scholarly concerns about choice in healthcare settings as pertaining to three main issues: the ‘responsibilisation of the citizen’, ‘patient vulnerability and needs’ and ‘risks associated with introducing choice into healthcare’. For instance, Rose (1999) argues that: ‘Individuals are now to be linked into a society through socially sanctioned consumption and responsible choice’ (1999: 166). Crucially for Rose, individual choice is often coupled with the discourse and process of responsibilisation, which shifts responsibility away from institutions towards individuals, while simultaneously ignoring individual capacity to be vulnerable.

These arguments and concepts are worthy of further examination in relation to biomedical research in contexts and populations with under-resourced healthcare systems. In the next section, the article turns to some of the sociological and anthropological examinations of choice in biomedical research participation in deprived and marginalised populations.

### Choosing biomedical research

Biomedical research provides certain parameters around levels of care and benefit to participants currently enrolled in research. These parameters are made explicit in information sheets provided to prospective research participants. The information provided also establishes the form that ethical values take in the conduct of research which is generally promotes conditions deemed to be favourable to autonomous decision-making and duties. If individuals wish to participate in research then they must provide their consent.

The consenting process has been critiqued by several sociologists who have extended concerns about neoliberalism in healthcare provision to its role in the conduct of biomedical research (Yasar, 2010). They have argued that biomedical research places even greater emphasis on ideas of choice than those observed in healthcare provision by being governed almost exclusively by a libertarian ethical imperative, i.e. the ethical imperative is to provide individual choice and promote autonomous decision-making (Peterson, 2014). Consequently, other potential ethical approaches to biomedical research such as virtue ethics are deemed uneconomic, paternalistic and inoperable.

In the last 10 years, sociologists such as Fisher (2009) and Abadie (2010) have investigated and provided insightful accounts of what it means to ‘choose’ to be a healthy ‘volunteer’ in American clinical trials. For Abadie, research contracts transform marginalised, unemployed and deprived populations into employees for clinical trials or in his words ‘professional guinea-pigs’. Individuals choosing research as their ‘employment’ do so by accepting the risks involved. The individual is also responsible for examining the terms and conditions outlined by research institutions. If they agree to be a research participant, then they also accept its risks and responsibilities. This position reinforces the aforementioned argument presented by Rose on individual responsibilisation. Furthermore, Du Gay argues that institutional emphasis on choice is of instrumental value because it ‘creates a distance between the decisions of formal political institutions and other social actors [and] conceives of these actors as subjects of responsibility, autonomy and choice, and seeks to act on them through shaping and utilising their freedom’ (2000: 168). From this position, emphasising individual choice allows for a shift from institutional to individual responsibilities. The institutional weight placed on choice also shapes the focus of ethical regulation of research. The ethical regulation of research then becomes limited to ensuring that potential research participants produce autonomous decisions (Fisher, 2013). Ethical concerns about the quality of the options available, whether individuals should treat their bodies as a means to an end and notions of bodily integrity are rendered paternalistic and foreclosed, as the logic of choice dominates. For these reasons, scholars have problematised choice in studies of research participation, treating the notion of volunteering as highly questionable in resource-poor contexts.

### Choice beyond researcher–participant interactions

Fisher (2007, 2013) aims to move the examination of choice beyond accounts of dyadic researcher–participant interaction to the socioeconomic forces which make research participation among the American poor, without universal access to healthcare, one of the most amenable forms of healthcare and employment. Drawing on scholarly contributions from Farmer (1996; Farmer et al., 2004) and labour studies more generally (see Reiman, 2012), Fisher (2013) proposes that the concept of ‘structural coercion’ allows for a more nuanced analysis of choice and decisions within resource-poor contexts.

Fisher (2013) mobilises the concept of structural coercion in critiquing the regulatory focus on overt coercion and undue influence to enrol in research in the researcher–participant relationship. She argues that ‘structural coercion’ is necessary to attend to the narrow focus on coercion because it:

… no longer privileges individuals, [but] can operate without any threat of overt violence … In contrast to conventional forms of coercion, the threat of violence is not tied directly to the research opportunity. Indeed, potential participants may turn to research in order to mitigate the threat of structural violence. (2013: 360)

Structural coercion does not suggest that research participants lack autonomy or understanding of the features of their enrolment in biomedical research, as proposed in arguments of therapeutic misconception (Appelbaum et al., 1987). Rather, possessing knowledge and agency does not mitigate structural factors which construct research participation as the most viable option for providing an income and healthcare.

This discussion about structural coercion mirrors the concept of ‘soft coercion’ which focuses on the impact of structural factors on research participation in Sub-Saharan African contexts (Oyedeji et al., 2010). Soft coercion is concerned with the notion of choice for research participants with long-term chronic conditions such as HIV infection (Ukpong and Akanni, 2008). These participants need to ensure continued lifelong and consistent access to drugs. This situation severely constrains their choice to withdraw from biomedical research that offers them access to drugs, services and care which would have been otherwise inaccessible. The concept of soft coercion makes an important contribution to the literature because it is concerned not only with the role of poverty in shaping choice in enrolling into research but also with factors which maintain participation as well as other considerations, such as power relationships and imbalances between doctors as researchers and patients as research participants. In what follows, I will attempt a further examination of these arguments in relation to biomedical research conducted in the Global South.

### Offshoring and the globalisation of choice

A significant development in the conduct of biomedical research in the last 30 years has been the ‘offshoring’ of research designed in the Global North to be conducted in the Global South (Petryna, 2009; Rajan, 2006). This offshoring of research has been enabled by globalisation and the deployment of technologies permitting whole or partial phases of research to operate in multiple regions around the world. Offshoring has also been assisted by ideas that biomedical research can and should operate by a financial imperative to reduce costs while maximising profits. Consequently, public and private research institutions have sought out cheaper and invariably poorer countries as attractive and financially rewarding research locations (Crane, 2013).

However, scholarly attention has also highlighted how market forces and competition between countries to host clinical trials determine where offshoring occurs. A cost-effective location includes ready-to-recruit populations, large labour pools of educated potential employees and a relatively stable political environment (Cooper and Waldby, 2014). Offshoring overheads means less expenditure on participants in terms of reimbursements for their time and transport costs, staff salaries and the costs involved in instances of serious adverse events (Crane, 2010; Folayan and Allman, 2011). Such varying standards across different locations have produced what Petryna (2005) refers to as ‘ethical variability’ (i.e. unstandardised ethical practice) between countries.

Despite the differences in healthcare provision and regulatory infrastructure between countries, the logic of choice still prevails as a justification and explanation for where research takes place. Governments and countries are presented by research organisations as choosing to host biomedical research within their borders. Local scientific and ethics committees legitimise this process in reviewing research protocols for their scientific merit and safeguarding participants’ autonomy through an appropriately informed consenting process. These committees might stress the need for local language translation or other methodological and technical issues but they are expected to ‘rubber stamp’ research approved in the Global North, not assess the overall contribution of particular studies to strengthening local structural issues (Kass et al., 2007; Moran et al., 2009; Peterson, 2014). Yet, there are very few fora where the impact of these issues on research participation can be addressed holistically and in the absence of an open discussion they can be argued not to exist (Biehl and Petryna, 2013). In this way, as argued by Du Gay (2000), Northern institutions and funders can strategically distance themselves from the responsibilities involved in addressing local structural concerns by either understating the effects and extent of structural factors or accepting that there are structural concerns but emphasising choice at the level of government and individual research participants to host research and accept its terms and conditions.

The remaining sections of this article are concerned with exploring how ideas of choice are practised in biomedical research in resource-poor contexts in developing countries. In so doing I will foreground the positions and perspectives of frontline research staff. From the literature it appears that one of the effects of the emphasis on choice, predicated on the researcher–participant relationship, is that it diminishes the importance of structural factors. This article demonstrates the implications of this decontextualised approach to choice in Sub-Saharan African contexts. I begin by presenting the methods and then examining choice in practice. I foreground the views of frontline research staff and their position that despite the rhetoric, few socioeconomically deprived individuals have the choice of equally viable healthcare options, when the resources of international research institutions are considered alongside those of resource-poor government health facilities. Therefore, from the position of frontline research staff, such as fieldworkers, potential research participants have an empty choice. Finally, what research participants and frontline staff regard as the function of the emphasis on choice in biomedical research in resource-poor contexts is discussed.

## Methods

The arguments presented in this article are based on qualitative and ethnographic examinations of biomedical research in two locations in East and West African contexts. The data were collected intermittently for extensive periods between 2007 and 2014.

The research institutions involved had numerous similarities and differences. For instance, they conducted a range of biomedical research programmes examining diseases including HIV/AIDS and tuberculosis and malaria. These programmes formed part of longstanding international collaborations between African research centres and European and American organisations and funders. In most cases, the principal investigators and funders were European and American. The frontline research staff, including research nurses, doctors and fieldworkers, were African and usually recruited in close proximity to the research institutions. The main difference between institutions was the contractual basis on which staff were recruited. In one institution, the frontline research staff were kept on longer employment contracts which meant that they were then eligible for other benefits. This was also the institution that involved greater numbers of African principal investigators.

The data in this article were drawn from multiple studies. During the fieldwork, the everyday practices of locally recruited frontline research workers such as fieldworkers and nurses were examined. While the specific aims of these studies varied, the key questions focused attention on capturing the perspectives and positions of frontline research staff on what constituted ethics. During these discussions, the theme of choice emerged as an important concept in different perspectives on research conduct.

### Ethical approval

This examination of the views of frontline research staff involved gaining ethics approval from a number of different institutions. Ethical approval was sought from seven different institutions which included the author’s UK-based academic institution and national ethical boards in the respective countries. Furthermore, permission was sought from frontline staff and their line managers (e.g. principal investigators) involved in research projects.

In order to underscore the general relevance of the findings presented in this article, the individuals and institutions involved have been anonymised. Furthermore, to preserve the anonymity of these sites, they will be referred to as Location A and Location B.

### Research methods

This current analysis involved several different methods, including observations, in-depth interviews, focus group discussions and a graphic elucidation technique. Frontline research staff, including research nurses, fieldworkers and research doctors, were accompanied during their working and non-working lives. This involved observing data collection interactions between staff and research participants. While their roles varied, the close proximity of frontline research staff to research participants was a common feature of their work. In seeking to gain the views of frontline research staff of their everyday lives and on ethics, this article accepts that there are multiple ways to perceive, understand and discuss the world (e.g. Stanley and Wise, 1993). Within this approach, informants’ accounts of ethics are not regarded as objective but rather they were seen as valuable in illuminating a particular view and position on ethics.

In addition, frontline research staff were involved in focus group discussions (FGDs) to gain insights into the dominant themes about research practices. These served as an introduction to the aims and were conducted only with research staff. All FGDs were audio recorded and transcribed and were approximately 90 minutes in length. There were 11 FGDs in total (6 in Location A and 5 in Location B).

Additionally, in-depth interviews were used to explore significant themes arising from the FGDs and observations. Over the seven-year period I conducted 42 in-depth interviews, which were longer than the FGDs, ranging from 45 minutes to three hours. They were conducted in a variety of places chosen by interviewees: cafes, hotel bars and private homes. These interviews generally occurred outside working hours and were less structured than the FGDs with more focus on eliciting personal insights. During this type of interview, particular attention was given to contrasting views to those expressed in the FGDs.

### Graphic elicitation technique

In recent years, graphic elicitation techniques have become increasingly popular with social scientists seeking to gain visual representations of their informants’ positions on a range of subjects (Crilly et al., 2006). Generally, this method either requires researchers to produce and present illustrations to their participants or involves participants producing illustrations (Galman, 2009). Instead, in this study an illustrator was employed who was unfamiliar with these locations. The illustrator created visual representations for this study based on the author’s observations, conversations and data gained during interactions with frontline research staff. These illustrations were shown to frontline research staff to elicit their responses, and then used to modify representations.

Discussions of illustrations generally took place informally within a small group. From such discussions, the modification of illustrations continued until there was a general consensus among frontline research staff that the illustrations produced were representative of their views. These illustrations allowed for perspectives and interpretations of this sensitive topic to be anonymously presented (Bagnoli, 2009). So while the illustrations aimed to represent everyday events, they also protected the identities of this cadre of research staff. The illustrations informed future interviews with frontline research staff and senior researchers, which proved valuable in interpreting and analysing the data. For these reasons, this method was used as a key source of data, informing the reflexive and analytical processes involved in the production of data.

## Findings

### Structural factors shaping the choice of research participation

**Figure 1.**
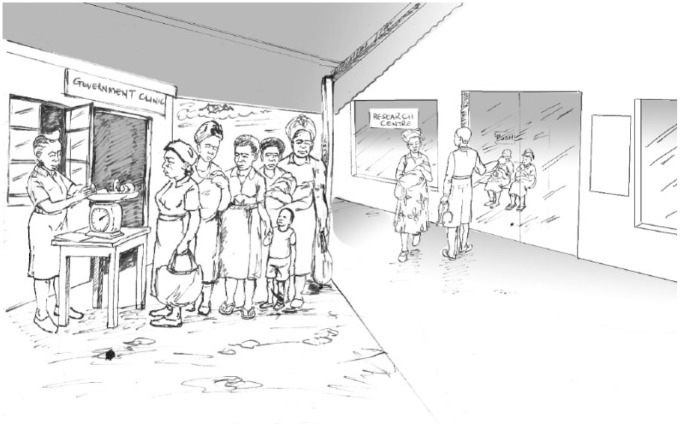
The empty choice.

Figure 1 was developed with fieldworkers and other frontline research staff to depict a familiar scene observed on numerous occasions. It demonstrates the overall argument of this article that potential research participants are presented with an ‘empty choice’ in relation to their participation.

The illustration shows a mother and a young child about to join a queue of women waiting to be seen at a government health facility. Figure 1 makes clear that the resources of the health facility, in terms of staff and access to modern equipment, are limited and demand for its services exceeds supply. The government service aims to immunise and weigh children as part of a national health programme. As is often the case, many mothers are motivated to receive this service and have arrived early. Despite their best efforts they are often in long queues taking several hours.

When they are finally seen they are often greeted by an exhausted healthcare provider. The figure shows one nurse immunising babies with physical and emotional stress evident on her face. Furthermore, it is not uncommon for women to find having endured the queue that the vaccines have run out and they are asked to return on another occasion. For these women, it is not only that their children have not been immunised but they must incur the opportunity cost of missed activities which could have benefitted their household.

Figure 1 shows that in contrast to the queue of women at the government facility, mothers are seen immediately at the paediatric research clinic which operates within the same grounds. The research clinic looks modern and more comfortable. The staff developing the illustration emphasised the differing levels of comfort experienced by research participants compared to non-participants. This is depicted by the chairs provided for participants. However, frontline research staff considered that the most attractive feature of research was the caring attitude shown to women and children and an acknowledgement of the importance of their time. Speed and efficiency were vital for ensuring that the women could continue with their daily income-generating activities and with caring for their children and families.

Figure 1 was developed iteratively. In an early version, the author suggested including a fieldworker asking a mother joining the queue whether she wanted to enrol her child in biomedical research. Fieldworkers recruiting participants do approach mothers to request that they consider their children’s participation in research. However, this suggestion was taken by staff involved in the illustration to suggest they were being coercive in requesting participation. The illustration was altered to foreground structural factors shaping the choice of research participation, to demonstrate that concerns for individual acts of coercion or undue inducement were misplaced.

### Being a research participant or a patient in a government facility

The frontline research staff involved in developing Figure 1 emphasised differences in staff expertise. It was suggested that a more accurate description of the everyday reality meant greater access to doctors in research than in government facilities. Therefore, potential research participants are presented with more than a choice between the physical space, efficiency and levels of comfort – they also had the potential to access professionals with greater expertise.

In the following quote, a research doctor compares his experience in a government hospital with his current position in an international research institution. The quote demonstrates his comparative positions but it also puts into contrast the experiences of patients in a government facility and those as research participants. The research doctor explains that:

When I worked … [in the government health facility] You don’t want to open up and be too free and friendly because then will come more problems than you can deal with ‘doctor I can’t afford this and that thing … medicines … food … can you help me?’ and you cannot help …

… Simple, simple things you know would make the difference in someone’s health you can’t do…

… when we tell them at the end of studies or if they do not fulfil our [recruitment] criteria that they have to go back to [government facility] they look at you like you are sending them away to die …That’s why working here [at the research facility] is so much easier for me. I have all the equipment I need and I can help … (Research doctor, in-depth interview, Location A)

This quote provides an insight into what choice looks like in practice when different resources are being considered by research participants. It demonstrates choice cannot be decontextualised and uncoupled from notions of suffering. According to the doctor quoted, a research participant’s choice is shaped by the competing sources of healthcare. If, as described here, the government hospital is associated with maltreatment and death, asking individuals to choose between this and having access to healthcare through biomedical research participation was tantamount to presenting them with an empty choice.

### Your consent or your life

One of the classic examples of the empty choice is the ‘Your money or your life’ decision. Here, the parameters are framed so narrowly that an individual effectively has no choice. In this way, while an individual can be regarded as acting between choices as an autonomous agent, their choice is severely constrained by the inequality in the options they have been given. For many frontline research staff, the poor were being presented with similar types of choices when considering research participation. In this quote a research nurse contextualises the concept of choice:

When you look at those women who are in our studies … they are really from the poorest here … No one made them do it … but on another look you really wonder ‘Have they chosen?’ … it’s life or death out there if they are not with us … I have seen it … so what choice do they really have? (Research nurse, in-depth interview, Location B)

This research nurse openly questions the idea of choice when the viable options are constrained by structural factors. This quote is valuable in foregrounding notions of suffering and vulnerability as factors external to the researcher–participant dyad but which frame the dynamics and freedom of choice in that relationship. The empty choices faced by potential research participants actually do not preclude them from choosing not to enrol in biomedical research. This quote emphasises that individuals are making decisions about research participation ‘the alternative of declining participation is always available to them’. However, the alternatives in terms of the quality and convenience of healthcare are so disproportionate that it can appear that they have no choice.

This quote also elucidates one of the consequences of institutions distancing themselves from responsibilities related to the research context. The above quotation provided by the research nurse demonstrates that often, the responsibility for examining the nature of the decisions made by prospective research participants is left to the frontline research nurse. It is the nurse who questions the impact of structural factors on the decision-making of deprived individuals. This is important because such examinations of the quality of the options available to individual research participants was rarely address at institutional level.

### The function of choice

Given the critiques of the current emphasis on choice among frontline research staff operating in resource-limited contexts, it was valuable to explore what was considered the function of emphasising choice over other (ethical) values such as care, dignity and justice. Numerous explanations were proposed. Some argued that the focus on choice was a strategy by funders and research institutions to justify and legitimatise research, while knowing that research participants in resource-limited contexts had little or no choice. Others suggest that an emphasis on choice as an ethical practice reflects a European approach and that there was something fundamentally different about ethical conduct in contexts with different infrastructures, histories and culture. While not everyone was in agreement with these positions, choice as practised in these contexts was largely seen to be incompatible with considering ideas of fairness and justice, that the very act of placing individuals in the position where they had to make such choices was unjust. Furthermore, it was a generally held view that choice had some form of instrumental purpose rather than being promoted for its own sake.

The following quote obtained from a research doctor was selected because it encapsulates many of the key points presented by frontline research staff when discussing choice. Here she discusses her position on the emphasis given to individual choice in the enrolment of research participants:

… it’s my personal opinion that this whole business is a farce … You [are] looking at a mother who has lost 1, 2, 3 children before they are 5 [years old] and for what? For a lack of a bed net or antibiotics and you ask her if she wants to join research with her new born? OK, so I have to go through with this process to make sure she is not being coerced and yes that’s important. But for who? Not for this mother and not for me. I don’t have to do anything to coerce her! … Just stop and look at her existence. The question should be what can I do to make her life easier?

… sometimes I think that all this emphasis on autonomy and coercion is guilt because of the Nazis and I want to say ‘I’m not a Nazi! I’m capable of mistakes but I’m just a doctor who has been trained to do the best for people!’ Let’s find another way to make research fair … and ethically done than just relying on whether someone has given their consent. (Research doctor, in-depth interview, Location B)

This research doctor’s description of freedom of choice as a ‘farce’ echoes Nordgren’s suggestion of empty words or the performance of choice. In this instance, emphasising choice was seen as being disingenuous and ignores individual suffering and vulnerability. Furthermore, she criticises the association with historical atrocities as a means of shaping the conduct and ethical imperative of research in terms of autonomy and choice rather than a holistic approach to the best interests of research participants.

This research doctor is clearly frustrated and her account provides an insight into some of the challenges involved in conducting research in such conditions. Earlier in this article, the notion of offshoring was introduced. This quote suggests that not only are research overheads offshored but that some important moral evaluations are also outsourced to the research encounter. It is left to frontline staff to manage the lack of healthcare options for participants while at the same time performing the existence of choice.

### Research participants as employees

In the following quote, a principal investigator takes a different view on choice in making research equitable. He advocates exposing research to greater market forces, making participants research employees, and more competition between research projects based on levels of payment:

I really don’t know what could be an alternative … people need to choose to be in research … There’s no way you could deprive rich people in America and Europe the right to choose so why should you take it away from poor Africans! We should choose what is best for us … We need to treat participants fairly … Stop expecting Africans to work for research for free … They should be given a salary based on local wages … so they are being paid fairly for their time … What they choose to spend the money on is up to them … That way we are developing here … (Principal investigator, in-depth interview, Location B)

This quote demonstrates that not all frontline research staff held the same view on choice. This principal investigator saw that the only solution to the current problems identified in the practice of neoliberalism and libertarian ethical values was more, not less, emphasis on choice. From this position, research participants should be treated as employees and market forces should dictate their salaries, employment conditions and research projects with the most favourable conditions. However, when considering research participants as employees, a fieldworker contends that:

Giving people the choice to be part of research really doesn’t stop bad things from happening to them. It just makes those bad things their fault for not asking the right questions or reading the fine print. (Fieldworker, FGD, Location A)

This fieldworker draws attention to the strategy of emphasising individual choice to distance researchers and institutions from the obligations to be solely responsible for research participants.

## Discussion

The current emphasis on choice is argued to be economically and ethically important to the conduct of biomedical research. Economically, research informed by market forces and neoliberal policies has led to a reduction in costs, as institutions in the Global North search for institutions and governments in the Global South to host research. Ethically, the key paradigm of biomedical research over the last 60 years has been the emphasis on individual choice as an ethical imperative. Ethical research is one where participants have entered into it voluntarily.

The literature examining choice in healthcare provision has been valuable in providing a critique of choice from mostly North American and European contexts. The literature has focused attention on the everyday consequences of macro-economic neoliberal ideology in the health and well-being of patients. In examinations of choice in biomedical research, the concepts of structural and soft coercion have been important in attempting to move discussion away from the researcher–participant dyad to foregrounding contextual and structural factors which constrain healthcare options to such an extent that attempts to enact a choice in research enrolment becomes a perfunctory performance.

It has been shown in this article that many critiques of the emphasis on choice in European and North American healthcare settings are also applicable to biomedical research, specifically the ways in which the responsibility for managing these structural factors is often consigned to the researcher–participant dyad. In the absence of institutional action, researchers are left with the moral responsibility and burden of performing choice while knowing that not being involved in research renders healthcare, drugs and qualified staff inaccessible to prospective participants and research participants with chronic conditions in some contexts. In this way, the concept of offshoring of biomedical research is given further analytical depth when discussed alongside ideas such as responsibilisation. Collectively, these concepts and the accounts of frontline research staff reveal that a key feature in the practice of offshoring of biomedical research to resource-limited contexts, where prospective participants are presented with an empty choice, is the ‘moral outsourcing’ of responsibilities to be managed in the frontline researcher–participant dyad.

This article has challenged the current tendancy to place moral weight on choice. It has argued that prospective participants are often faced with a choice of whether or not they enrol in research. This article also argues that insufficient attention is given to the quality of their options in terms of the choices with which they are presented. This article has introduced the concept of the ‘empty choice’ as a means of moving the discussion from the rhetoric of choice to being concerned with the quality of the options available to individuals within their everyday lives.

Furthermore, this article makes a contribution to the literature by presenting data collected from those working within biomedical research who are tasked with enacting choice. From the position of frontline research staff operating in under-resourced healthcare systems in Sub-Saharan African contexts, structural factors cannot be uncoupled from the choice presented to individuals; they permeate the options and decisions involved in research participation. Furthermore, they argue that possessing knowledge and agency does not mitigate the weight of these structural factors acting on individuals. Frontline research staff have argued that prospective research participants often participate in biomedical research because the benefits are clear. While staff perform the role of providing choice to individuals, they are fully aware of the lack of alternatives for the poor wishing to prioritise their health. Based on the accounts presented by frontline research workers in this article, it is clear that if institutions in the Global North operating in the Global South are invested in promoting not only informed choice but free and autonomous consent in research participation, they ought to play a much greater role in strengthening the health systems in the countries in which research is conducted.
